# Neural Progenitor Cells Derived from Human Embryonic Stem Cells as an Origin of Dopaminergic Neurons

**DOI:** 10.1155/2015/647437

**Published:** 2015-04-30

**Authors:** Parinya Noisa, Taneli Raivio, Wei Cui

**Affiliations:** ^1^School of Biotechnology, Institute of Agricultural Technology, Suranaree University of Technology, Nakhon Ratchasima 30000, Thailand; ^2^Institute of Biomedicine, Department of Physiology, University of Helsinki, 00290 Helsinki, Finland; ^3^Children's Hospital, Helsinki University Central Hospital, 00290 Helsinki, Finland; ^4^Institute of Reproductive and Developmental Biology, Department of Surgery and Cancer, Faculty of Medicine, Imperial College London, Hammersmith Campus, London W12 0NN, UK

## Abstract

Human embryonic stem cells (hESCs) are able to proliferate *in vitro* indefinitely without losing their ability to differentiate into multiple cell types upon exposure to appropriate signals. Particularly, the ability of hESCs to differentiate into neuronal subtypes is fundamental to develop cell-based therapies for several neurodegenerative disorders, such as Alzheimer's disease, Huntington's disease, and Parkinson's disease. In this study, we differentiated hESCs to dopaminergic neurons via an intermediate stage, neural progenitor cells (NPCs). hESCs were induced to neural progenitor cells by Dorsomorphin, a small molecule that inhibits BMP signalling. The resulting neural progenitor cells exhibited neural bipolarity with high expression of neural progenitor genes and possessed multipotential differentiation ability. CBF1 and bFGF responsiveness of these hES-NP cells suggested their similarity to embryonic neural progenitor cells. A substantial number of dopaminergic neurons were derived from hES-NP cells upon supplementation of FGF8 and SHH, key dopaminergic neuron inducers. Importantly, multiple markers of midbrain neurons were detected, including *NURR1, PITX3*, and *EN1*, suggesting that hESC-derived dopaminergic neurons attained the midbrain identity. Altogether, this work underscored the generation of neural progenitor cells that retain the properties of embryonic neural progenitor cells. These cells will serve as an unlimited source for the derivation of dopaminergic neurons, which might be applicable for treating patients with Parkinson's disease.

## 1. Introduction

Human embryonic stem cells (hESCs) have the ability to renew and differentiate into multiple cell types of the three germ layers upon exposure to appropriate signals [1]. Considerably, the differentiation of hESCs in culture appears to follow the hierarchical set of signals that regulate embryonic development in the generation of the germ layers and specific cell types [2]. Besides, hESCs provide a valid model to understand the complex signaling interactions during early embryonic development. Parkinson's disease (PD) is an incurable and progressive condition characterized by the continuing degeneration of dopaminergic (DA) neurons in the midbrain substantia nigra. The specific loss of DA neurons makes cell-based replacement a promising strategy to cure PD patients. Previous clinical studies involving transplantation of embryonic ventral mesencephalic tissues in PD patients have proved the feasibility of cell-based therapy in this disease; however, this approach is restricted due to the shortage of donor tissues and ethical concerns [3]. Thus, the possibility of generating DA neurons from hESCs offers an alternative source of cells for regenerating DA neurons in the midbrain of PD patients [4].

Derivation of specific cell types from hESCs becomes more attractive due to the recent approval of clinical trials using hESC derivatives to treat several neurodegenerative diseases, such as spinal cord injury and age-related macular degeneration. It has been shown that neural progenitor cells can be differentiated from hESCs and that they exhibit broad cellular spectrum with respect to developmental and lineage specification [5, 6]. Differentiation of hESCs in an adherent culture reveals striking similarity between* in vitro* differentiation and* in vivo* embryonic formation of neuroectoderm [7]. BMP antagonism has been considered as the central initiating event in neural induction and neuroepithelial specification that occurs as* a default pathway* [8]. The antineurogenic effect of BMPs is thought to be, at least, partly mediated via induction of gene expression for* ID1* and* ID3,* helix-loop-helix transcription factors important for proper neural development [9, 10]. ID proteins act as dominant-negative regulators that interfere with the transcriptional activities of proneural proteins in neural progenitor cells [11]. Neural progenitor cells have been successfully generated from hESCs by applying Noggin, one of the BMP antagonists; nonetheless, the high cost of recombinant Noggin hampers the applicability of this protocol [5]. Alternatively, small molecules blocking both BMP and Activin/nodal pathways have also been used in inducing neural differentiation from human pluripotent stem cells. However, this dual inhibition of SMAD signaling is often accompanied with a massive cell death, which might limit the application when a large number of cells are needed [6, 12]. It is noted that neural differentiation is a dynamic process. Various stages of neural derivatives are generated, which show distinct properties [7]. During the differentiation of hES cells, neural progenitors transit from a predominantly neuronal state into one with increased gliogenic potential. This phenomenon resembles what is found in embryonic neurogenesis [13].

Following neural lineage specification, it is critical to establish the positional information that could induce phenotypes of a particular type of neurons. Gradients of signaling molecules can regionally specify a population of neural progenitor cells [14]. This achievement will accelerate the progress of potential uses of hESCs in cell replacement for neurodegenerative diseases, including Parkinson's disease. DA neurons have been derived from human pluripotent stem cells by the forced expression of key developmental genes for neuronal fate, such as* LMX1a* and* ATOH1* [15, 16]. Although this transgene strategy offers a high-yield production of DA neurons, the contamination of exogenous DNA hinders the applicability of the resulting neurons in PD patients. It is known that sonic hedgehog (SHH) and fibroblast growth factor 8 (FGF8) regulate the initial stages of DA neuronal development to specify DA progenitors in the medial ventral area of the midbrain [17]. These extrinsic factors control a number of key transcription factors such as* NGN2*,* MASH1*,* FOXA2*,* LMX1a, LMX1b*,* NURR1*, and* PITX3* that are crucial for DA neurogenesis [18–20]. The supplementation of SHH and FGF8 should guide neural progenitor cells to become DA neurons* in vitro*.

In this study, we optimized the differentiation system to induce hESCs into neural lineage by a single small molecule Dorsomorphin. The resulting neural progenitor cells were then directed to DA neurons by using the two key patterning molecules, FGF8 and SHH.

## 2. Results

### 2.1. Dorsomorphin Is a Strong Neural Lineage Inducer

**Figure 1:**
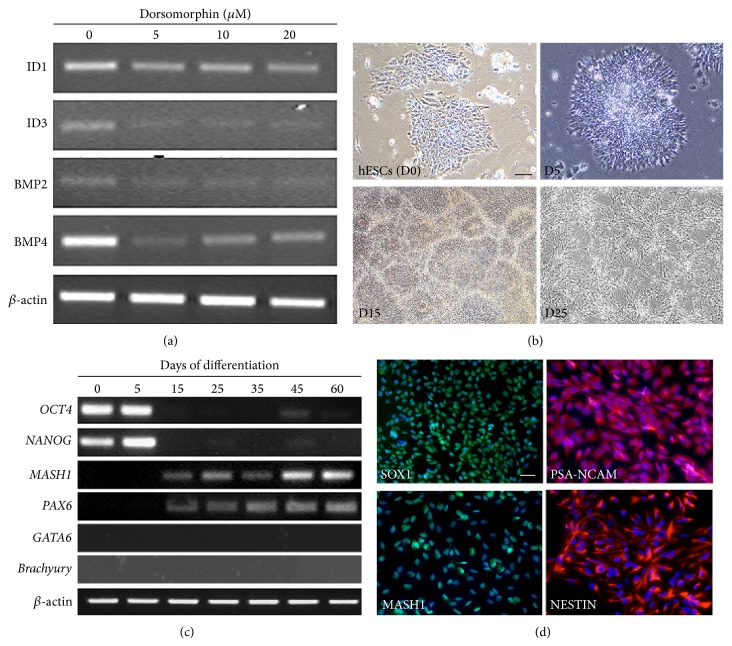
Differentiation of neural progenitor cells from hESCs by using small molecules. (a) Dorsomorphin, a small molecule inhibiting BMP signaling, was tested at various concentrations. (b) Morphology of hESCs transformed during the differentiation. Neural rosette structures appeared after 15 days of the differentiation prior to the generation of bipolar neural progenitor cells. (c) Gene expression analysis at different days of the differentiation. Pluripotency genes were downregulated, while neural progenitor genes were increased along the differentiation.* GATA6* and* Brachyury* were not detected during neural differentiation. (d) Neural progenitor cells homogeneously expressed SOX1, PSA-NCAM, and NESTIN.

hESCs can be efficiently induced into neural lineage by a potent BMP inhibitor, such as Noggin [5]. However, continuous supplementation of recombinant Noggin is costly since the initiation of neural lineage from hESCs takes several days. In this study, we used Dorsomorphin, a small chemical compound, to specifically inhibit BMP signaling pathway [21]. The effect of different concentrations (5 *μ*M, 10 *μ*M, and 20 *μ*M) of Dorsomorphin on the expression of BMP effector genes, including* ID1* and* ID3*, as well as BMP ligands,* BMP2* and* BMP4*, was first tested. After 5 days, expression of these genes was downregulated (Figure 1(a)) even with the 5 *μ*M Dorsomorphin, and, therefore, this concentration was employed in subsequent experiments. hESCs transformed from epithelial structures (D0; Figure 1(b)) and appeared as distinct morphology after the differentiation process began (day 5; Figure 1(b)). A trivial number of death cells were observed. Thereafter, cells were spontaneously patterned to form neural rosette structures, in which the cell morphology was similar to that of neuroepithelium (day 15; Figure 1(b)). A similar morphological change was also observed in human induced pluripotent stem cells (data not shown). During this initiation stage, BMP inhibitor, Dorsomorphin, is required to block extraembryonic endoderm differentiation. After the formation of neural rosette structures, Dorsomorphin was replaced by 10 ng/mL bFGF in the neural differentiation medium and the cells exhibited bipolar structure, a typical morphology of neural progenitor cells, and we named these cells as hES-NP cells (day 25; Figure 1(b)). Gene expression was examined along the differentiation process of hES-NP cells by RT-PCR. Pluripotent genes,* OCT4* and* NANOG*, were downregulated, coinciding with the upregulation of neural lineage markers,* MASH1* and* PAX6* (Figure 1(c)). Expression of other lineage markers, such as* GATA6* and* Brachyury*, was not detected, indicating that no other cell types were generated under the current differentiation system (Figure 1(c)). Immunocytochemistry results revealed homogeneous expression of neural progenitor markers, including SOX1, MASH1, and NESTIN (Figure 1(d)). Interestingly, hES-NP cells expressed PSA-NCAM, a marker of intermediate neural progenitor cells [22] (Figure 1(d)). This set of markers is considered as diagnostic molecules for embryonic neural stem cells [23].

### 2.2. hES-NP Cells Are Multipotent

**Figure 2:**
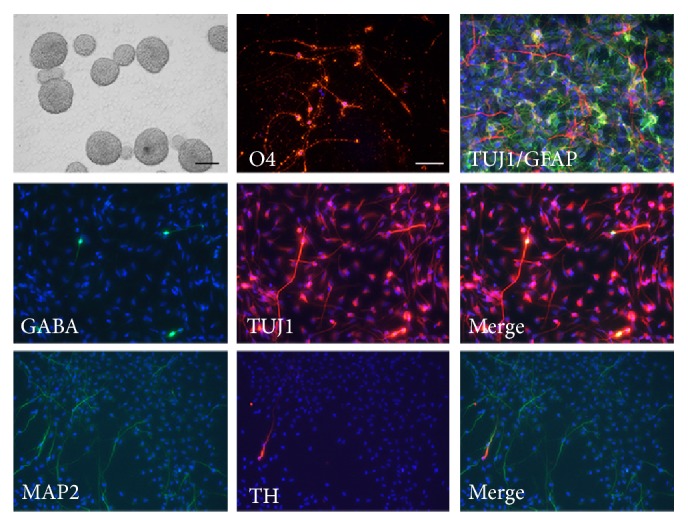
Differentiation potential of neural progenitor cells derived from human embryonic stem cells. In order to induce terminal differentiation, neural progenitor cells were let to form neurospheres for 7 days. The cells were then replated, immunostained, and found to give rise to several cell types, such as oligodendrocytes (O4), GABAergic neurons (GABA), and dopaminergic neurons (TH). TUJ1-positive neurons (red) and GFAP-positive glial cells were codetected in differentiating culture.

One of the key characteristics of neural progenitor cells is multipotency, that is, the ability to give rise to several types of neuronal cells. To examine the differentiation potency of hES-NP cells, the cells were subjected to further differentiation. Neurospheres of hES-NP cells were formed in low attachment dish with N2B27 medium, supplemented with 1% fetal calf serum (FCS). After 2 weeks, hES-NP cells were plated onto coverslips for immunocytochemistry. Differentiated hES-NP cells were positive for neuronal marker *β*-tubulin III and astrocyte marker GFAP, while markers for oligodendrocytes were absent (Figure 2). To induce oligodendrocytes from hES-NP cells, cells were cultured in N2 medium, supplemented with PDGF, Forskolin, platelet-derived growth factor (PDGF), triiodothyronine (T3), and ascorbic acid, as described in Materials and Methods. Oligodendrocytes were rarely generated, consistent with previous studies [24, 25]. In addition to simple neuronal differentiation, hES-NP cells could give rise to specific neuronal subtypes, for example, GABAergic and DA neurons even at low frequency (Figure 2). These results confirmed the multipotency of hES-NP cells.

### 2.3. hES-NP Cells Have Low CBF1 Responsiveness

**Figure 3:**
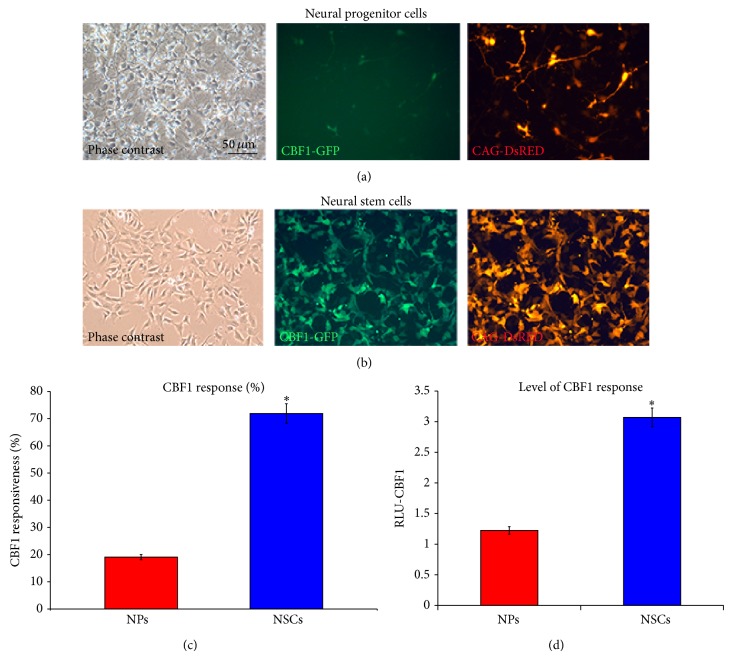
Low CBF1 response of neural progenitor cells. ((a)–(c)) CBF1 response of cells was measured by transient transfection of GFP-reporter plasmid. ((a) and (b)) Phase contrast and fluorescence images of neural progenitor cells and neural progenitor/stem cells, respectively. Scale bar represented 50 *μ*M for all images. (c) Histogram showed percentage of CBF1-responsive cells. (d) Histogram presented luciferase activity controlled by CBF1. Three independent experiments were performed with a standard deviation bar and ∗ represented *p* < 0.005 (Student's* t*-test).

Notch signaling is well known to regulate neural lineage initiation, neural stem cell maintenance, and cell fate determination [26]. The activation of canonical Notch signalling pathway through C-promoter binding factor 1 (CBF1) plays a critical role in the signaling transduction [27]. Differential response to CBF1 is an important feature to distinguish the state of neural progenitor cells from neural stem cells, which is independent of activation of Notch receptors [28]. During neural differentiation process of hES cells, various stages of neural derivatives have emerged, including early neural progenitors and GFAP-positive neural stem cells [7]. To elucidate that hES-NP cells resemble embryonic neural progenitor cells, CBF1 responsiveness was evaluated, and hES-neural stem cells, as described in Materials and Methods, were used as a comparison. CBF1 responsiveness was demonstrated by transient transfection of CBF1-responsive reporter plasmids, in which the reporter genes, GFP and luciferase, are driven by promoter containing 4 CBF1-responsive elements. The result showed that most of hES-neural stem cells (~70%) expressed CBF1-GFP while only small population (~20%) of the hES-NP cells did (Figures 3(a)–3(c)). Furthermore, luciferase assay displayed a similar pattern that hES-neural stem cells exhibited 3 times higher CBF1-luciferase activity than that of hES-NP cells (Figure 3(d)). These results demonstrated that hES-NP cells had minimal response to CBF1, whereas hES-neural stem responds to it well. This evidence suggested that hES-NP cells resembled embryonic neural progenitor cells, found early during neurogenesis.

### 2.4. hES-NP Cells Are Mitotically Responsive to bFGF

**Figure 4:**
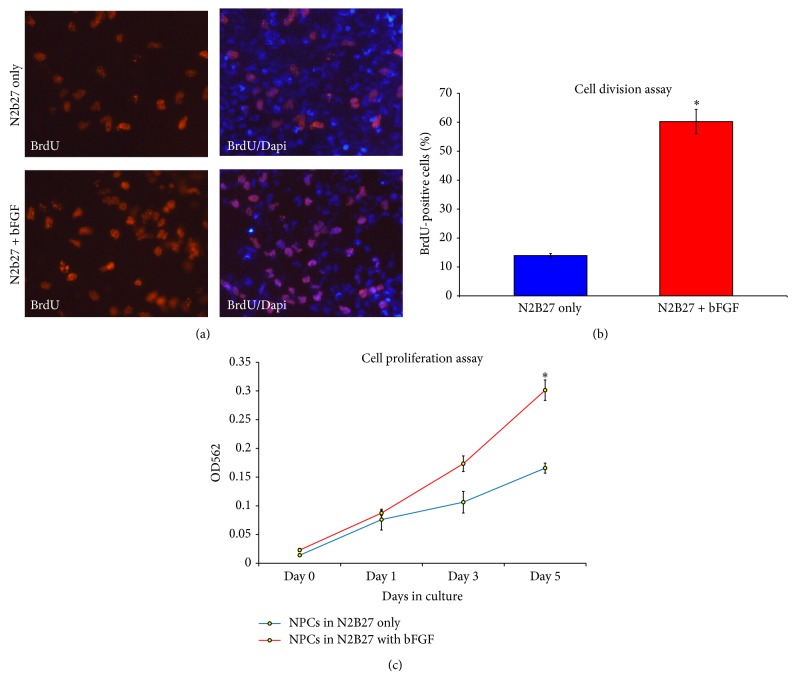
Proliferation capacity of neural progenitor cells derived from human embryonic stem cells. bFGF can promote proliferation of neural progenitor cells. (a) BrdU assay showed the increased proliferation of neural progenitor cells upon the addition of bFGF. (b) The number of BrdU-positive cells was significantly higher than in the control cells. (c) MTT assay confirmed the effect of bFGF on neural progenitor cell proliferation. Data are presented as mean ± s.d. (*n* = 3). ∗ represents *p* < 0.05 by Student's *t*-test.

During embryonic neurodevelopment, proliferation of neural progenitor cells is regulated by a variety of soluble factors, including bFGF and EGF. Neural progenitor cells require bFGF and EGF at different stages of brain development. Survival and proliferation of embryonic progenitor cells appear to be regulated by bFGF and predisposed toward neuronal differentiation [29]. On the other hand, later in the development, EGF-responsive progenitor cells are the predominant species and are likely to become more glial lineages [30]. It is interesting to examine the mitogenic response of hES-NPCs, which might reflect their developmental state of an* in vivo* counterpart. Mitogenic response of hES-NP cells was examined by BrdU cell division assay and MTT cell proliferation assay (Figure 4). It appeared that hES-NPC population responded well to bFGF supplementation (Figure 4(a)). hES-NP cells significantly increased the percentage of dividing cells, from 13.2% ± 0.8% to 61.7% ± 6.3%, upon the addition of 10 ng/mL bFGF (Figure 4(b)). The mitogenic effect of bFGF on hES-NP cells was confirmed by MTT cell proliferation assay. Addition of 10 ng/mL bFGF significantly promoted the proliferation of hES-NP cells after 5 days of the experiment (Figure 4(c)).

### 2.5. Dopaminergic Neurons Can Be Derived from Neural Progenitor Cells

**Figure 5:**
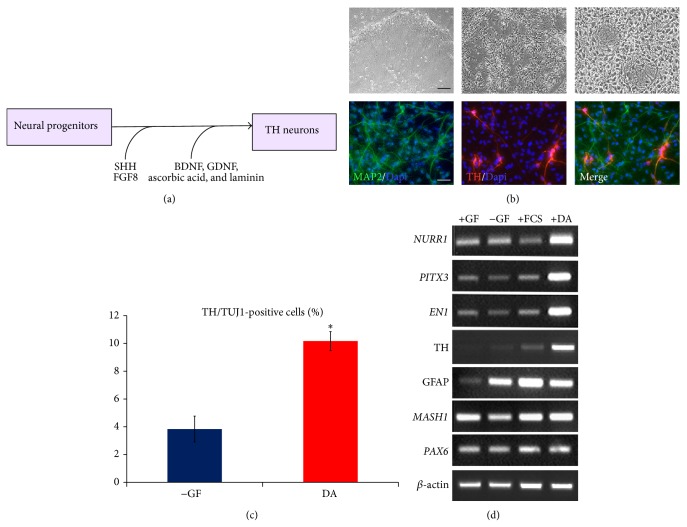
Direct dopaminergic neuron differentiation from neural progenitor cells. (a) Schematic of differentiation conditions for DA neurons from neural progenitors. (b) Phase contrast pictures showing morphological changes of cells during the differentiation. Cells were immunostained against MAP2 and TH, indicating dopaminergic neuron phenotypes. (c) The number of TH-positive neurons in DA-inducing condition (DA) was significantly greater than that without growth factor condition (−GF). Data are presented as mean ± s.d. (*n* = 3). ∗ represents *p* < 0.05 by Student's *t*-test. (d) Gene expression result suggested that differentiated neural progenitor cells in DA condition expressed midbrain dopaminergic neuron markers, including* NURR1*,* PITX3*, and* EN1*.

In addition to glial-neuron differentiation, hES-NP cells were examined for their differentiation into dopaminergic (DA) neurons by supplementing two developmental cues for DA neurogenesis, SHH and FGF8 [31–33]. Ventral midbrain/hindbrain fates of hES-NP cells were induced by exposure to FGF8 and SHH followed by treatment with neurotrophic factors, including BDNF, GDNF, ascorbic acid, and laminin (Figure 5(a)). When subjecting hES-NP cells to such differentiation regime, they showed mature neuronal structure and were positive for MAP2/TH immunostaining, hallmark characteristics of DA neurons (Figure 5(b)). A significant number of hES-NP cells became MAP2/TH-double positive DA neurons (10.6% ± 1.2%), while only 3.8% ± 1.3% of MAP2/TH-double positive cells were found in the control differentiating medium (without any growth factors) (Figure 5(c)). However, it is necessary to confirm that DA neurons generated in these experiments were midbrain-origin which are promisingly required for the treatment of Parkinson's disease. We examined the expression of molecular markers of midbrain in DA neurons derived from hES-NP cells at various conditions: (1) prior to the induction toward DA neurons (+GF), (2) without the supplementation of growth factors (−GF), (3) in the presence of 1% fetal calf serum (+FCS), and (4) DA neuron-inducing conditions (DA) as stated above. The pan-neuronal genes,* MASH1* and* PAX6*, did not show variable expression among the different culture conditions; however,* NURR1*,* PITX1*, and* EN1* were clearly upregulated in the DA conditions (Figure 5(d)). This verified that hES-NP cells could serve as an expandable source for DA neurons. The adjustment of regional identities via morphogens mimics environmental cues found in the embryonic brains.

## 3. Discussion

Neurogenesis in mammals is a complex process involving many different cell types that differentiate asynchronously. Our understanding about it is far from complete. In addition to the complicated process, the relatively small number of neuronal cells that can be readily obtained from embryonic brain makes the study of the neural development extremely difficult. hESCs are derived from early embryos and have the potential to generate all the cell types in the nervous system; therefore, they should be a suitable* in vitro* model for studying neural development [34, 35]. In early development, neural progenitor cells transit from NESTIN-positive single layer neuroepithelial cells to S100*β*-positive radial glial cells and eventually give rise to GFAP-positive adult neural stem cells [36]. Several studies have reported the generation of mouse ESC-derived progenitor cells with radial glial properties that can be propagated in the presence of bFGF [37, 38]. hES-NP cells can also be propagated in the presence of bFGF, liberated from any requirement for a specific cellular niche. The cells exhibited multiple prominent characteristics: (1) stability in a long-term culture (>100 days), (2) ability to produce neurons and glia, and (3) expression of neural progenitor markers, indicating the appropriateness of our culture system to maintain neural progenitor identity. This study highlights that hES-NP cells are similar to embryonic neural progenitors in several ways, including the detection of PSA-NCAM expression and mitogenic responsiveness to bFGF [23, 29]. In addition, the differential use of Notch signaling, in particular with respect to CBF1 activation, is a mechanism used to distinguish the state of cells, whether they are neural progenitors or neural stem cells [28]. Here we showed that CBF1 responsiveness of hES-NP cells was considerably low, comparing to hES-neural stem cells. This data is in accordance with the growth factor responsiveness, showing that hES-NP cells mitotically responded to bFGF (Figure 4). These results altogether implied that hES-NP cells are similar, at certain degree, to embryonic neural progenitor cells. Moreover, this result indicates that the activation or blockade of canonical Notch-CBF1 cascade may direct expression of different target genes, which determine the neural/glial potency of neural progenitor cells [39]. The capacity of hES-NP cells to respond to patterning cues, which are here FGF8 and SHH, emphasizes the potential use of these cells for regenerative medicine.

Several available protocols showed that targeting SMAD signaling can induce hESCs toward neural lineage by suppression of alternative embryonic germ layers [6, 12, 40, 41]. However, the simultaneous blockade of BMP- and Activin/TGF-*β*-mediated SMAD signaling pathways results in gross cell death at the initial phase of the differentiation. The inhibition of BMP signaling alone by Noggin showed efficient neural differentiation; however, this routine supplementation of recombinant protein is costly [5]. In this study, we modified the protocol for neural differentiation from hESCs by using a single small molecule Dorsomorphin that can inhibit BMP signaling and found that this compound is sufficient to convert hESCs into neural progenitor cells. As compared to Noggin, Dorsomorphin has a wider action on BMP signalling because it blocks BMP signalling at receptor level, rather than binding and inhibiting a distinct set of BMP proteins as Noggin does [21, 42]. Besides ALK receptors, Dorsomorphin also inhibits the activity of several other kinase enzymes and thereby modulates cell physiology through multiple signalling pathways that might facilitate neural differentiation of hESCs [21]. In contrast to other studies, we blocked only BMP signalling, but not Activin/TGF-*β* pathway with SB431542. It has been reported that Activin/TGF-*β* pathway is important for neuronal cell development and survival [43, 44]. Moreover, instead of N2 supplement, we used N2B27 as an additive to the basal medium. B27 supplement is comprised of an enriched combination of insulin and multiple antioxidant substances that may promote the proliferation and survival of neural progenitor cells [45, 46].

Since hES-NP cells are uncommitted and proliferative, they could serve as a scalable source for the production of various types of neurons and glial cells for toxicant and pharmaceutical screening and, ultimately, for cell-based replacement therapy. It will be important to determine hES-NPC differentiation capacity to generate different neuronal subtypes* in vitro* and* in vivo* and whether or how the cells can contribute functional reconstruction in diseased animals. Here we presented that a substantial number of DA neurons could be obtained from hES-NP cells following the exposure of FGF8 and SHH. Differentiation efficiency of dopaminergic neurons could also be enhanced by using a recombinant midbrain factor, for instance, LMX1A [47]. Nevertheless, further development of culture systems, combining with tissue engineering technology, will offer an improved microenvironment and increase differentiation efficiency of hES-NP cells toward DA neurons [48]. The clinical grade differentiation system to produce DA neurons should be developed in order to translate these cells into clinical uses [49]. Besides DA neurons, other neuronal subtypes should be able to obtain from hES-NP cells when appropriate conditions are applied such as cholinergic neurons, GABAergic neurons, and glutaminergic neurons. The development of neuronal subtype specification protocols will expand the application of hES-NP cells to other neurological diseases. Altogether, this study provided a system for generating neural progenitor cells from hESCs by using a single BMP inhibitor Dorsomorphin. Moreover, the resulting hES-NP cells could serve as an expandable source for DA neurons production, which could be applied for various purposes.

## 4. Materials and Methods

### 4.1. Culture of Human Pluripotent Stem Cell

H7 and H9 hESCs (WiCell) and Hell11.4 iPSCs were cultured in Geltrex-coated plates with Stem Pro complete medium (Invitrogen) and routinely propagated with combination of 0.02% PBS/EDTA treatment and mechanic dissociation.

### 4.2. Differentiation and Culturing Conditions of Neural Progenitor Cells Derived from hESCs

Confluent H9 hESCs were split with 0.02% EDTA/PBS (Sigma) in 1 : 5 ratios into culture dishes coated with Geltrex (Invitrogen) and cultured in N2B27 medium (all supplements from Gibco), supplemented with 5 *μ*M Dorsomorphin (Sigma). At this stage, cells were defined as passage 1 (P1) and the cell was called hES-NP cells. In the neural differentiation, the cells were consistently cultured in the Geltrex-coated culture plates. Cells of P1 and P2 were split by 0.02% EDTA/PBS (Sigma) into small clumps, similar to hESC culture, and continuously cultured in N2B27 medium plus Dorsomorphin. From P3, cells were dissociated into single cells by TrypLE express (Gibco), and Dorsomorphin could be withdrawn from N2B27 medium with the addition of 10 ng/mL bFGF. The cells were able to survive in long-term culture. hES-NP cells gradually change their morphology and gene expression profile, when cultured over passage 30, and we termed these cells as hES-neural stem cells. To induce postmitotic cell types, hES-NP cells were cultured in the basal medium without growth factors for 2 weeks.

### 4.3. Differentiation of Oligodendrocytes from hES-NP Cells

hES-NP cells were plated on PLL/laminin-coated dishes and the neural stem cell expansion medium (N2B27) was replaced by DMEM/F12 supplemented with N2 (N2 medium). Cells were proliferated in the presence of 10 ng/mL PDGF and 10 mM Forskolin for 7 days to support a glial precursor stage. Further differentiation was induced by a 7-day-growth culture in the presence of 10 ng/mL PDGF, 30 ng/mL 3, 3, 5-triiodothyronine (T3), and 200 *μ*M ascorbic acid (AA).

### 4.4. Differentiation of DA Neurons from hES-NP Cells

Neural progenitor cells were seeded in PLL/Lam-coated culture dishes and induced with sonic hedgehog (400 ng/mL), FGF8 (100 ng/mL), and ascorbic acid (160 *μ*M) for 2 weeks and then followed by brain-derived neurotrophic factor (BDNF) (20 ng/mL), glial cell line-derived neurotrophic factor (GDNF) (20 ng/mL), ascorbic acid (160 *μ*M), and laminin (0.5 *μ*g/mL) for another week. RT-PCR and immunocytochemistry were performed to characterize phenotypes of the cells.

### 4.5. Reverse Transcription and Polymerase Chain Reaction (RT-PCR)

Total RNA was extracted using RNA Spin II (Macherey-Nagel) by following the manufacturer's instructions. First-strand cDNA was synthesized from 2 *μ*g total RNA by SuperScript III reverse transcriptase (Invitrogen) with oligo dT primer (Invitrogen) in 20 *μ*L volume. cDNA solution was diluted 1/10 in dH_2_O and 2 *μ*L was used for each PCR reaction. The list of target gene primers was supplied.

### 4.6. Immunocytochemistry

Cells were fixed at room temperature with 4% paraformaldehyde for 10 minutes. Nonspecific proteins were blocked by incubation in phosphate buffered saline (PBS) containing 10% fetal bovine serum (Sigma) and 0.1% Triton X-100 for one hour at room temperature. The cells were then treated with primary antibodies overnight at 4°C. After washing with PBS, the cells were incubated with fluorescence-conjugated secondary antibody for 45 minutes and finally mounted to cover slip with Vectashield mounting medium with DAPI (Vectorlabs). The following primary antibodies were used in this study: monoclonal antibodies against TH, NESTIN (1 : 200, all from Chemicon), and *β*-tubulin III (Sigma, 1 : 1000); rabbit polyclonal against Musashi-1 (1 : 200), MAP2 (1 : 1000, all from Chemicon), SOX1 (1 : 100), SOX2 (1 : 50; all from Abcam), and glial fibrillary acidic protein (GFAP; 1 : 500; Dako Cytomation). Secondary antibodies used were goat anti-mouse immunoglobulin Alex Fluor 568 and goat anti-rabbit Alex Fluor 488 (all at 1 : 400; Molecular Probes).

### 4.7. BrdU Cell Division Assay

Cells cultured on PLL/laminin precoated cover slip in 24-well plates were incubated with BrdU at a final concentration of 10 *μ*M at 37°C for 2 hours. After washing with PBS, cells were fixed for 10 minutes in freshly prepared 4% paraformaldehyde. After washing again, cells were incubated with 2 M HCl at 37°C for 1 hour to denature the DNA, followed by incubation for 1 hour in blocking buffer containing 10% goat serum, 0.5% Triton X-100. The cells were then treated with monoclonal antibody against BrdU for 1 hour and secondary antibody for 30 minutes in dark with PBS washing in between. The slides were mounted and left to dry before visualization.

### 4.8. MTT Cell Proliferation Assay

MTT stock solution was prepared at a concentration of 5 mg/mL in PBS and stored at 4°C. 25 *μ*L of MTT solution was added to each well and the cells were incubated for 2 hours to allow the reaction to occur. Then, 100 *μ*L MTT lysis buffer (10% SDS) was added to each well, and the plates incubated overnight to ensure that the hypermetabolic cellular states will not interfere with the final reading. MTT conversion has determined the absorbance/optical density (OD) at 562 nm. Lysis controls were used to give background control reading.

### 4.9. Luciferase Activity Assay

Dual-luciferase reporter (DLR) assay system was used to determine luciferase activity following the manufacturer's protocol. Briefly, to prepare stock solution, luciferase assay reagent II (LAR II) was prepared by resuspending the lyophilized substrate in 10 mL of the supplied buffer II. LAR II solution was kept at −80°C until used. Stop&Glo reagent was diluted just prior to the assay for a desired volume with the provided buffer. Cells were transfected for 48 hours with the firefly reporter and* Renilla* luciferase vectors and then lysed by 500 *μ*L passive lysis buffer (PLB) before being transferred to a 1.5 mL tube. 100 *μ*L of LAR II was predispensed into the appropriate number of luminometer plates to complete the desired number of DLR assays. 20 *μ*L of cell lysate was carefully transferred to the luminometer plate containing LAR II, mixed by pipetting and initiated reading by the luminometer. Next, 100 *μ*L of prepared Stop&Glo reagent was added to the previous mixture, mixed by pipetting and initiated reading. Calculation of luciferase activity was determined by relatively comparing the activity of firefly and* Renilla* luciferase.

## Supplementary Material

Supplementary table 1: is a list of primer sequences used in this study
Supplementary table 2: is information of antibody used in this study.
