# Supplementary material for: Neural Progenitor Cells Derived from Human Embryonic Stem Cells as an Origin of Dopaminergic Neurons
Source: Stem Cells Int. 2015 Apr 30;2015:647437. doi: 10.1155/2015/647437 (PMC4430666; doi:10.1155/2015/647437)
Supplement: Supplementary file 1 — Supplementary table 1: is a list of primer sequences used in this study Supplementary table 2: is information of antibody used in this study. [file 647437.f1.pdf]

Supplementary Table 1. List of primers used in this study

Gene	Forward primers	Reverse primers
**BMP2**	CTTCTAGCGTTGCTGCTTCC	TGCTTGCATTCTGATTCACC
**BMP4**	ACCTGAGACGGGGAAGAAAA	TTAAAGAGGAAACGAAAAGCA
**b-Actin**	TCACCACCACGGCCGAGCG	TCTCCTTCTGCATCCTGTCG
**ID1**	ACCCTGCCCCAGAACCGCAAG	TTGTTCTCCCTCAGATCCG
**ID3**	ACTCACTCCCCAGCATGAAG	AAGCTCCTTTTGTCGTTGGA
**OCT4**	GACAACAATGAAAATCTTCAGGAGA	TTCTGGCGCCGGTTACAGAACCA
**NANOG**	AGCCTCTACTCTTCCTACCACC	TCCAAAGCAGCCTCCAAGTC
**MASH1**	TCGCACAACCTGCATCTTTA	CTTTTGCACACAAGCTGCAT
**PAX6**	AACAGACACAGCCCTCACAAACA	CGGGAACTTGAACTGGAACTGAC
**GATA6**	CCATGACTCCAACTTCCACC	ACGGAGGACGTGACTTCGGC
**Brachyury**	TGCTTCCCTGAGACCCAGTT	GATCACTTCTTTCCTTTGCATCAAG
**NURR1**	TACTGCCGATTTCAGAAGTGC	AATCAATCCATTCCCCAAAGC
**PITX3**	ACTAGGCCCTACACACAG	AGTCCGCGCACGTTTATT
**EN1**	TGGTCAAAACTGACTCGCAGCA	TCTCGTCTTTGTCCTGGACCGT
**TH**	GTACTTCGTGCGCCTCGAGGTG	GCGTGGACAGCTTCTCAATTTC
**GFAP**	TCATCGCTCAGGAGGTCC TT	CTGTTGCCAGAGATGGAGGTT

Supplementary Table 2. List of antibodies used in this study

Antibody	Company, Cat. No.	Dilution
**SOX1**	Abcam, AB22572	1:100
**BrdU**	DSHB, G3G4	1:1000
**MASH1**	ABCAM, AB74065	1:1000
**Nestin**	Chemicon, MAB5326	1:200
**TUJ1**	Sigma, T8660	1:1000
**GFAP**	DAKO, Z0334	1:500
**GABA**	Sigma, A2050	1:200
**MAP2**	Chemicon, AB5622	1:1000
**TH**	Chemicon, MAB318	1:200
**PSA-NCAM**	Chemicon, MAB5324	1:200
